# Microbiological Quality and Safety of Fresh Turkey Meat at Retail Level, Including the Presence of ESBL-Producing *Enterobacteriaceae* and Methicillin-Resistant *S. aureus*

**DOI:** 10.3390/foods12061274

**Published:** 2023-03-16

**Authors:** Alba Martínez-Laorden, Celia Arraiz-Fernández, Elena González-Fandos

**Affiliations:** Department of Food Technology, CIVA Research Center, University of La Rioja, Madre de Dios, 26006 Logroño, Spain

**Keywords:** poultry, food safety, ESBL, MRSA, meat, *E. coli*, *K. pneumoniae*, enterococci, staphylococci, antibiotic residues, multi-resistance

## Abstract

The aim of this work was to study the microbiological safety and quality of marketed fresh turkey meat, with special emphasis on methicillin-resistant *S. aureus*, ESBL-producing *E. coli*, and *K. pneumoniae*. A total of 51 fresh turkey meat samples were collected at retail level in Spain. Mesophile, *Pseudomonas* spp., enterococci, *Enterobacteriaceae*, and staphylococci counts were 5.10 ± 1.36, 3.17 ± 0.87, 2.03 ± 0.58, 3.18 ± 1.00, and 2.52 ± 0.96 log CFU/g, respectively. Neither *Campylobacter* spp. nor *Clostridium perfringens* was detected in any sample. ESBL-producing *K. pneumoniae* and *E. coli* were detected in 22 (43.14%), and three (5.88%) samples, respectively, all of which were multi-resistant. Resistance to antimicrobials of category A (monobactams, and glycilcyclines) and category B (cephalosporins of third or fourth generation, polymixins, and quinolones), according to the European Medicine Agency classification, was found among the *Enterobacteriaceae* isolates. *S. aureus* and methicillin-resistant *S. aureus* were detected in nine (17.65%) and four samples (7.84%), respectively. Resistance to antimicrobials of category A (mupirocin, linezolid, rifampicin, and vancomycin) and category B (cephalosporins of third- or fourth generation) was found among *S. aureus*, coagulase-negative staphylococci, and *M. caseolyticus* isolates.

## 1. Introduction

Consumption of turkey meat has increased in recent years due to its characteristics of low cost, high protein content, and low fat content (1.21%) (lower than the fat content of chicken) [1]. However, turkey meat has been involved in outbreaks of *Salmonella*, *Staphylococcus aureus*, *Campylobacter* spp., *Clostridium perfringens*, and *Listeria monocytogenes* [2].

The microbiological contamination of poultry meat is influenced by the settings under which animals are reared, transported, slaughtered, processed, and stored [3,4,5]. The microbiota of poultry meat is composed of different types of bacteria, including *Pseudomonas* spp., *Enterobacteriaceae*, *Staphylococcus* spp., *Brochotrix thermosphacta*, *Acinetobacter*, lactic acid bacteria, and *Aeromonas* spp. [5,6,7]. The main spoilage bacteria associated with poultry meat are *Pseudomonas* spp., lactic acid bacteria, *B. thermosphacta*, and *Enterobacteriaceae* [6].

The bacterial communities found in poultry meat comprise spoilage bacteria and, in some cases, foodborne pathogens such as *Salmonella*, *Campylobacter* spp., *Staphylococcus aureus*, *Clostridium perfringens*, and *Listeria monocytogenes* [5,7]. Poultry meat can be contaminated by bacteria present in the gastrointestinal tract (*Lactobacillus* spp., *entecocci*, *Clostridium* spp., *Ochrobacterium* spp., *Corynebacterium* spp., and *Enterobacteriaceae*) and in the skin, feathers, and feet of birds (*Staphylococcus* spp., *Corynebacterium* spp., *Propionibacterium* spp., and *Acinetobacter Moraxellla*) [8,9,10]. Another relevant source of contamination is the processing environment (*Pseudomonas* spp., *Sphingobacterium* spp., *Acinetobacter* spp., *Vagococcus* spp., *Carnobacterium* spp., *Lactobacillus* spp., *Leuconostoc* spp., and *Listeria* spp.) [5,8,11].

The majority of studies on the microbiology of fresh poultry meat have been undertaken on chicken meat [6,12,13,14,15,16]. Less information is available on fresh turkey meat [17,18]. Therefore, it is of interest to know the microbiota present in fresh turkey meat, as well as the populations of relevant groups in the poultry sector, such as *Enterobacteriaceae*, enterococci, staphylococci, and *Pseudomonas* spp. It is worth noting that the main source of microbiological contamination of carcasses in slaughterhouses is of fecal origin; therefore, *Enterobacteriaceae* and *E. coli* are considered as useful hygiene indicators [19]. On the other hand, enterococci are commensals in the gut of animals; thus, the contamination of turkey carcasses with enterococci can occur during slaughter if hygienic standards are low [20]. 

Staphylococci are frequent inhabitants of poultry skin [21]. While some species of the genera *Staphylococcus*, such as *S. aureus*, are recognized pathogens, other species are considered as commensals [9]. Since *Pseudomonas* spp. are among the most important spoilage bacteria in poultry [13], it is relevant to know the populations and species that are present in fresh turkey meat.

Currently, antimicrobial resistance is considered a major public health issue [22]. The spread of extended-spectrum β-lactamase (ESBL) and carbapenemase-producing *Enterobacteriaceae* is of particular interest [23]. Various works have shown that *Escherichia coli* isolated from turkey meat has a significant level of antimicrobial resistance [24]. Furthermore, extended-spectrum β-lactamase (ESBL)-producing *Klebsiella pneumoniae* and *E. coli* have been detected in turkey meat [25,26]. Methicillin-resistant *S. aureus* (MRSA) has been isolated from turkey meat [27]. This bacterium has often been associated with hospital-acquired infections [28]. Consequently, there is major interest in the role of meat in spreading antimicrobial resistance [23], especially in the case of ESBL-producing *Enterobacteriaceae* and methicillin-resistant *S. aureus*.

The aim of this work was to study the microbiological safety and quality of marketed fresh turkey meat, with special emphasis on methicillin-resistant *S. aureus*, ESBL-producing *K. pneumoniae*, and *E. coli*.

## 2. Materials and Methods

### 2.1. Turkey Meat Samples and Microbiological Analysis

A total of 51 fresh turkey meat samples were purchased in Logroño (La Rioja, Spain) from 10 different retailers that were representative of a variety of trade models. The samples were collected between January 2020 and January 2021. The number of samples of each commercial brand was determined according to the place-of-purchase data [29]. All the samples were produced in Spain. Fourteen samples were collected in two different hypermarkets (HA and HB), 35 were collected in seven different supermarkets (SA, SB, SC, SD, SE, SF, and SG) and two were collected in traditional shops (TAs).

The 51 meat samples were evaluated by the ultra-performance liquid chromatography quadrupole time of flight (UPLC–QTOF) method to detect antibiotic residues, as indicated in an earlier study [26]. Doxycycline was found in one sample at levels of 6.6 µg/kg. Antibiotic residues were not detected in the other 50 samples [30].

For the initial microbiological analysis, 10 g of turkey meat were aseptically taken and homogenized using a masticator blender (IUL Instruments, Barcelona, Spain) for 2 min with 90 mL of sterile peptone water (0.1% *w*/*v*) (Oxoid, Basingstoke, Hampshire, UK). Decimal dilutions were carried out using the same diluent. The next microbiological analyses were then carried out for Mesophiles, *Pseudomonas* spp., enterococci, *Enterobacteriaceae*, staphylococci, *Campylobacter* spp., and *Clostridium perfringens*. Mesophile counts were determined on Plate Count agar (Scharlau, Barcelona, Spain) after incubation for 48 h at 30 °C. The enumeration of *Pseudomonas* spp. was conducted in a chromogenic agar for *Pseudomonas* (Scharlau) incubated for 72 h at 30 °C. Enterococci were evaluated on Kanamycin Esculin Azide agar (Scharlau) incubated at 37 °C for 48 h. *Enterobacteriaceae* counts were evaluated using MacConkey agar (Oxoid) incubated at 37 °C for 24 h. Staphylococci were evaluated on Mannitol Salt agar (Oxoid) incubated at 35 °C for 36 h. *Clostridium perfringens* was evaluated in Tryptose Sulphite Cycloserine agar (Merck, Darmstadt, Germany) incubated at 40 °C for 24 h under anaerobic conditions.

To determine the presence of *Campylobacter* spp., 10 g of turkey meat were homogenized for 2 min in 90 mL of Bolton broth (Oxoid) and incubated at 42 °C for 1 day in a microaerobic atmosphere, followed by streaking on Agar Brilliance CampyCount agar incubated at 42 °C for 2 days under microaerobic conditions.

In addition, a screening was performed to determine methicillin-resistant *S. aureus* and ESBL- and carbapenemase-producing *Enterobacteriaceae*. Two grams of turkey meat were placed in flasks containing 50.0 mL of Brain Heart Infusion (BHI) broth (Oxoid) and incubated at 37 °C for 24 h. For the screening of methicillin-resistant *S. aureus* (MRSA), after incubation, the samples were plated with the streak-plate method in chromID MRSA agar (BioMérieux, Lyon, France) and incubated at 37 °C for 24 h. Presumptive MRSA colonies were selected for further analysis. For the screening of ESBL- and carbapenemase-producing *Enterobacteriaceae*, after incubation, the samples were plated with the streak-plate method in chromID ESBL and chromID CARBA SMART agar (BioMérieux) and incubated at 37 °C for 24 h. Presumptive *Escherichia coli* and *Klebsiella pneumoniae* were selected, according to the manufacturer’s instructions, for further analysis.

### 2.2. Isolation and Identification

From each turkey meat sample and culture media five colonies of the highest dilution that generated growth were randomly selected and isolated. The morphology of suspected colonies was taken into consideration when specific media were used. Isolates were purified in Tryptone Soy agar (Scharlau) and Brain Heart Infusion broth (Scharlau). The purified isolates were maintained at −80 °C. Bacterial identification was carried out by a MALDI-TOF biotyper (Bruker, Daltonik, Bremen, Germany).

### 2.3. Phenotypic Confirmation of ESBL Producers

Further analyses were carried out with isolates from chromID ESBL identified by MADI-TOF as *K. pneumoniae* and *E. coli*. Phenotypic confirmation of these ESBL producers was performed using the disc-diffusion method according to the Clinical Laboratory Standards Institute’s guidelines [31]. Isolates from other media identified as *K. pneumoniae* and *E. coli* were also analyzed.

### 2.4. Phenotypic Confirmation of Methicillin Resistance of S. aureus

The methicillin resistance of *S. aureus* was confirmed in accordance with the Clinical Laboratory Standards Institute’s guidelines [31] by a diffusion-agar assay using cefoxitin (30 μg).

### 2.5. Resistance of E. coli and Klebsiella spp. Isolates

The antimicrobial susceptibility of *K. pneumoniae*, *K. oxytoca*, and *E. coli* isolates was evaluated against a panel of 35 antimicrobials using the disk-diffusion method on Mueller–Hinton agar. For *E. coli*, one strain was chosen for each different medium and sample. All the *K. pneumoniae* isolates were selected. The next antibiotic disks (Oxoid) used were amikacin (AK, 30 µg), amoxicillin-clavulanate (AUG, 20/10 µg), ampicillin (AMP, 10 µg), ampicillin-surbactam (SAM, 10/10 µg), aztreonam (ATM, 30 µg), cefepime (FEP, 30 µg), cefotaxime (CTX, 30 µg), cefoxitin (FOX, 30 µg), ceftazidime (CAZ, 30 µg), cefpodoxime (CPD, 10 µg), ceftriaxone (CRO, 30 µg), chloranphenicol (C, 30 µg), doripenem (DOR, 10 µg), ciprofloxacin (CIP, 5 µg), doxycycline (DO, 30 µg), enrofloxacin (ENR, 5 µg), ertapenem (ETP, 10 µg), gatifloxacin (GAT, 5 µg), gentamicin (CN, 10 µg), iminepem (IPM 10, µg), kanamycin (K, 30 µg), levofloxacin (LEV, 5 µg), meropenem (MEM 10, µg), minocycline (MH, 30 µg), nitrofurantoin (F, 300 µg), nalidixic acid (NA, 30 µg), norfloxacin (NOR, 5 µg), piperacillin (PRL, 100 µg), streptomycin S (10, µg), sulfadiazine (SUZ, 300 µg), trimethoprim-sulfamethoxazole (SXT 1.25:23.75 µg), trimethoprim (W, 5 µg), tigecycline (TGC, 15 µg), tetracycline (TE, 30 µg), and tobramycin (TOB, 10 µg). After incubation at 37 °C for 18 to 24 h, inhibition zones were measured and scored as resistant, intermediate (reduced susceptibility), or susceptible in accordance with the Clinical and Laboratory Standards Institute’s guidelines [31]. The resistance to colistin was determined by the dilution method, in accordance with to the CLSI’s guidelines [31].

### 2.6. Resistance of Macrococcus spp. and Staphylococci Isolates

The antimicrobial susceptibility of eight *Macrococcus caseolyticus* and 66 staphylococci isolated was tested against a panel of 29 antimicrobials using the disk-diffusion method on Mueller–Hinton agar. For each species identified, one strain was selected for each different medium and sample. The next antibiotic disks (Oxoid) used were amikacin (AK, 30 µg), ceftaroline (CPT, 30 µg), chloramphfenicol (C, 30 µg), ciprofloxacin (CIP, 5 µg), cefoxitin (FOX, 30 µg), clindamycin (CMN, 2 µg), fusidic acid (FAD, 10 µg), erythromycin (ERY, 15 µg), enrofloxacin (ENR, 5 µg), gatifloxacin (GAT, 5 µg), levofloxacin (LEV, 5 µg), kanamycin (K, 30 µg), lincomycine (MY, 15 µg), gentamicin (CN, 10 µg), linezolid (LZD, 30 µg), mupirocin (PUM, 200 µg), nitrofurantoin (F, 300 µg), minocycline (MH, 30 µg), norfloxacin (NOR, 5 µg), streptomycin (S, 10 UI), penicillin (P, 10 UI), sulfadiazine (SUZ, 300 µg), trimethoprim -sulfamethoxazole (SXT 1.25:23.75 µg), tedizolid (TZD, 2 µg), doxycycline (DO, 30 µg), tetracycline (TE, 30 µg), rifampicin (RD, 5 µg), tobramycin (TOB, 10 µg), tylosin (TY, 30 µg), trimethoprim (W, 5 µg), and vancomycin (VA, 30 µg). For *S. saprophyticus* and *S. aureus*, quinupristin-dalfopristin (QD, 15 µg) was also tested; in the case of *S. aureus*, benzylpenicillin (PNG, 1 UI) was also tested. After incubation at 37 °C for 18 to 24 h, inhibition zones were measured and scored as resistant, intermediate (reduced susceptibility), or susceptible in accordance with the Clinical and Laboratory Standards Institute’s guidelines [31]. For *M. caseolyticus*, the resistance breakpoints for *Staphylococcus* spp. were used as suggested by Cotting et al. [32].

### 2.7. Statistical Analysis

The microbial counts were changed to log CFU/g. Analysis of variance techniques using Duncan’s multiple range test was carried out to separate averages and evaluate the three factors that were investigated: microbial group, retailer, and month. The level of significance was determined at *p* < 0.05. All the tests were conducted with SPSS version 26 software (IBM SPSS Statistics).

## 3. Results

Mesophile counts were 5.10 ± 1.36 log CFU/g, with counts in the range 2.3–7.23 log CFU/g. Only two samples showed levels above 7 log CFU/g. No significant differences (*p* > 0.05) in mesophile counts were observed between samples from hypermarkets and those from supermarkets. Nevertheless, significantly lower counts (*p* < 0.05) of mesophiles were observed in samples from traditional shops than in those from supermarkets and hypermarkets. No significant differences (*p* > 0.05) in mesophile counts were found between samples from the two hypermarkets. Significantly lower counts (*p* < 0.05) of mesophiles were found in supermarket SD than in the other six supermarkets analyzed.

The bacteria identified from the Plate Count agar were mainly one rifampicin (RD, 5 µg) and actic acid bacteria (37.66%), followed by *Brochotrix thermosphacta* (22.94%) (Table 1). *Pseudomonas* spp., *Enterobacteriaceae*, *Micrococcaceae*, and enterococci were isolated to a lesser extent (9.09%, 8.23%, 7.79%, and 1.30%, respectively) (Table 1). In addition, *Chryseobacterium* spp., *Acinetobacter* spp., *Brevundimonas diminuta*, *Stenotrophomonas rhizophila*, *Wautersiella falsenii*, *Psychrobacter pulomonis*, *Microbacterium* spp., *Rhodococcus erythropolis*, and *Bacillus endophyticus* were isolated (Table 1). *P. fragi* was the predominant *Pseudomnas* spp. isolated from Plate Count agar (47.62%) (Table 1). The meat sample in which doxycycline was detected showed mesophile counts of 5.15 ± 0.01 log CFU/g, being the species identified as *Brochotrix thermosphacta Rhodococcus erythropolis*, *Microbacterium liquefaciens*, and *Microbacterium maritypicum*. *R. erythropolis* and *M. maritypicum* were not identified in any other sample, while *M. liquefaciens* was isolated in two other samples. No significant differences (*p* > 0.05) in mesophile counts were found between the doxycycline-positive sample and those that were negative. The doxycycline levels detected in the positive sample were below the maximum residue limits (MRLs) of antimicrobials in meat, as established by Regulation 37/2010 (100 µg/kg) [33].

*Pseudomonas* spp. counts below 1 log CFU/g were observed in 15 samples (29.41%). The other 36 samples (70.59%) showed counts between 2.00 log CFU/g and 5.02 log CFU/g, with an average number of 3.17 ± 0.87 log CFU/g. No significant differences (*p* > 0.05) in pseudomonas counts were found between samples from hypermarkets and those from supermarkets. Nevertheless, significantly lower counts (*p* < 0.05) of pseudomonas were observed in samples from traditional shops than in those from supermarkets and hypermarkets. No significant differences (*p* > 0.05) in pseudomonas counts were observed between samples taken in the two hypermarkets. Significantly lower counts (*p* < 0.05) of pseudomonas were found in supermarket SD than in the other six supermarkets analyzed.

*Pseudomonas* spp. distribution is shown in Table 2. *P. libanensis* (31%) and *P. extremorientalis* (14%) were the prevailing species, followed by *P. fluorescens* (12%). The meat sample in which doxycycline was detected showed *Pseudomonas* counts of 2.24 ± 0.24 log CFU/g, being the only species isolated, *P. rhodesiae.*

Enterococci counts below 1 log CFU/g were found in 12 samples (23.53%). The other 39 samples (76.47%) displayed counts between 1.30 log CFU/g and 3.28 log CFU/g, with an average number of 2.03 ± 0.59 log CFU/g. No significant differences (*p* > 0.05) in enterococci were observed between samples from hypermarkets and those from supermarkets. However, significantly lower counts (*p* < 0.05) of enterococci were found in samples from traditional shops than in those from supermarkets and hypermarkets. No significant differences (*p* > 0.05) in enterococci counts were found between samples taken in the two hypermarkets. The *Enterococcus* spp. distribution is shown in Table 3. *E faecium* was the prevailing enterococci (38.10%), followed by *E. faecalis* (23.81%) and *E. gallinarum* (16.67%). In addition, *Streptococcus gallolyticus* was isolated in 12 samples (23.53% of the samples analyzed).

*Enterobacteriaceae* counts below 1 log CFU/g were found in 13 samples (25.49%). The other 38 samples (74.51%) showed counts between 1.60 and 4.99, with an average number of 3.18 ± 1.00. No significant differences (*p* > 0.05) in *Enterobacteriaceae* counts were observed between samples from hypermarkets and those from supermarkets. However, significantly lower counts (*p* < 0.05) of *Enterobacteriaceae* were observed in samples from. traditional shops than in those from supermarkets. No significant differences (*p* > 0.05) in *Enterobacteriaceae* counts were found between samples taken in the two hypermarkets. Significantly lower counts (*p* < 0.05) of staphylococci were found in supermarkets SD, SE, SF, and SG than in supermarkets SA, SB, and SC. Table 4 shows the species distribution. *Serratia liquefaciens* was the dominant specie (16.42%), followed by *Hafnia alvei* (14.18%) and *Escherichia coli* (14.18%). In addition, *Klebsiella pneumoniae*, *Moellerella wisconcensis*, and *Yersinia enterocolitica* were isolated. The meat sample in which doxycycline was detected showed *Enterobacteriaceae* counts of 2.69 ± 0.09 log CFU/g, being *E. coli* (40%) and *K. pneumoniae* (60%) the only species isolated. *K. pneumoniae* was not isolated from MacConkey agar in any other sample.

Twenty-three of the 51 turkey samples were positive in chromID ESBL (45.1%). ESBL-producing *K. pneumoniae* and *E. coli* were detected in three and 23 samples, respectively. ESBL-producing *E*. *coli* were confirmed phenotypically in 22 of 23 samples, while all ESBL-producing *K. pneumoniae* were confirmed. Both ESBL-producing *K. pneumoniae* and ESBL-producing *E. coli* were isolated from the meat sample in which doxycycline was detected. The *K. pneumoniae* isolates obtained from MacConkey agar in the doxycycline-positive sample were the ESBL-producing phenotype, while the two isolates of *K. oxytoca* obtained from doxycycline-negative samples were ESBL-negative. However, none of the *E. coli* isolates obtained from MacConkey agar showed the ESBL phenotype, although some of the isolates were obtained from samples that were positive in chromID ESBL. Carbapenemase-producing *Enterobacteriaceae* were not recovered from the chromID CARBA SMART medium.

**Figure 1:**
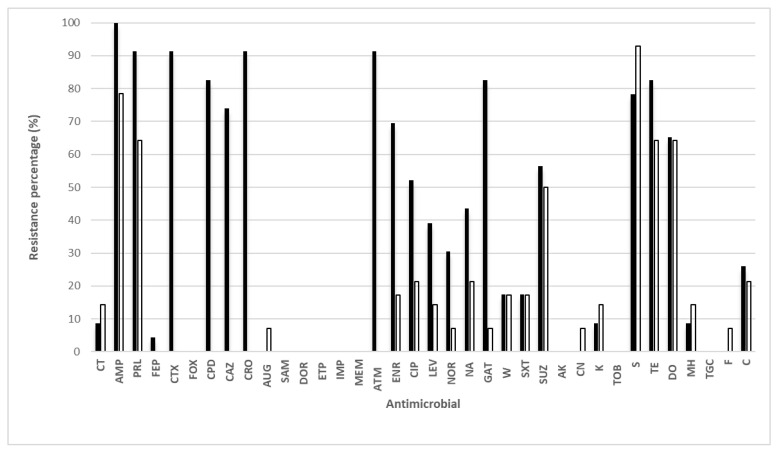
Antimicrobial resistance phenotype of *E. coli* isolated from turkey meat. CT: colistin, AMP: ampicillin, PRL: piperacillin. FEP: cefepim, CTX: cefotaxime, CPD: cefpodoxime, FOX: cefoxitin, CAZ: ceftazidime, CRO: ceftriaxone, AUG: amoxicillin-clavulanate, SAM: ampicilin + surfabactam, IMP: imipenem, DOR: doripenem, ETP: ertapenem, MEM meropenem, ATM: aztreonan, CIP: ciprofloxacin, ENR: enrofloxacin, LEV: levofloxacin, NOR: norfloxacin, NA: nalidixic acid GAT gatifloxacin, W: trimethoprim, SXT: trimethoprim- sulfamethoxzoale, SUZ: sulfadiazine, AK, amikacin, CN: gentamicin, K: kanamycin, TOB: tobramycin. S: streptomicin, TE: tetracycline, DO: doxycicline, MH: m, TGC: tigecycline, F: nitrofurantoin, C: chloramphenicol. ■ Isolates recovered from chromoID ESBL. □ Isolates recovered from MacConckey agar.

The antimicrobial resistance phenotype of *E. coli* isolates is displayed in Figure 1. All 23 *E. coli* isolates from chromID ESBL were multi-resistant (i.e., resistant to three or more antibiotic classes), with the highest rates of resistances to ampicillin (100%); piperacillin, ceftriaxone, and aztreonam (91.30%); cefpodoxime, gatifloxacin, and tetracycline (82.61); streptomycin (78.26%); cetftazidime (73.91%); and enrofloxacin (69.57%). For+ antimicrobial classes, the highest resistance corresponded to penicillins, cephalosporins, and monobactams. In addition, resistance to colistin was found (8.69%).

Of the 14 *E. coli* isolates from MacConkey agar, 71.43% were multi-resistant. The highest resistance rates were observed against streptomycin (92.86%); ampicillin (78.57%); and piperacillin, tetracycline, and doxycycline (64.29%). None of the isolates showed susceptibility to all of the 36 tested antibiotics.

Table 5 shows the antimicrobial resistance phenotype of multi-resistant *E. coli* isolated from turkey meat. Multi-resistant strains were isolated from samples obtained in supermarkets and hypermarkets. The highest number of multi-resistant *E. coli* was obtained in hypermarket HB (six isolates). However, no resistant *E. coli* strain was isolated from a traditional shop (TA).

The antimicrobial resistance phenotype of eight *K. pneumoniae* and two *K. oxytoca* isolates from turkey samples is shown in Table 6. All the *K. pneumoniae* isolates were multi-resistant, with the highest rates of resistance to ampicillin, piperacillin, cefpodoxime, ceftriaxone, enrofloxacin, ciprofloxacin, sulfadiazine, and streptomycin (100%); cefotaxime, cefepime, aztreonam, trimethoprim, trimethoprim-sulfamethoxazole, kanamycin, and tetracycline (87.5%); ceftazidime and tobramycin (75%); and doxycycline (62.5%). For antimicrobial classes, the highest resistance corresponded to penicillins, cephalosporins, monobactams aminoglycosides, tetracyclines, folate pathway-antagonists, and fluoroquinolones. In addition, resistance to colistin was found (50%). No resistance was observed against phenicoles. *K. pneumoinae* was only isolated from samples from one hypermarket (HA) and samples from two supermarkets (SA and SF).

Staphylococci counts were below 1 log CFU/g in 14 samples (27.45%). Counts ranged between 1.30 log CFU/g and 4.81 log CFU/g with an average number of 2.52 ± 0.96 log CFU/g. No significant differences (*p* > 0.05) in staphylococci counts were observed between samples from hypermarkets and those from supermarkets or a traditional shop. No significant differences (*p* > 0.05) in staphylococci counts were observed between samples taken in the two hypermarkets evaluated. Significantly lower counts (*p* < 0.05) of staphylococci were observed in supermarkets SD, SE, SF, and SG than in supermarkets SA, SB, and SC. Table 7 shows the *Staphylococcus* spp. distribution, with *S. saprophyticus* (31.45%) and *S. equorum* (13.7% being the dominant species. *S. aureus* was detected in nine samples (17.65%), being the fourth most often staphylococci isolated (8.1%). Methicillin-resistant *S. aureus* was detected in four samples (7.84%). *Macrococcus caseolyticus* was also identified (12.1%) (Table 6). The meat sample in which doxycycline was detected showed staphylococci counts of 1.3 ± 0.00 log CFU/g, being the only species identified, *S. warneri.*

Table 8 contains the antimicrobial resistance phenotype of *Macrococcus caseolyticus* isolates from turkey samples. It is worth noting that one strain (12.5%) was multi-resistant showing resistance to 11 antibiotics: lincomycine, mupirocin, fusidic acid. linezolid. penicillin, rifampicin, tedizolid, tylosin, vancomycin, erythromycin, and clindamycin. Only resistant *Macrococcus caseolyticus* was isolated from samples purchased in supermarkets SA, SB, SC, and SE.

Table 9 shows the antimicrobial resistance phenotype of methicillin-sensitive and methicillin-resistant *S. aureus* isolates from turkey meat. Most of the *S. aureus* isolates (88.89%) and all the methicillin-resistant isolates showed a multi-resistant phenotype. All the *S. aureus* isolates showed resistance to tetracycline, penicillin, and benzilpenicillin. Resistance to enrofloxacin was observed in 66.67% of the *S. aureus* isolates. Resistance to amikacin, chloramphenicol, kanamycin, mupirocin, tobramycin, ceftaroline, gentamycin, quinupristin-dalfopristin, rifampicin, and fusidic acid was only observed in 25–50% of the methicillin-resistant isolates, while resistance to clindamycine, erythromycin and tylosine was observe in 75% of these isolates. All the *S. aureus* isolates were susceptible to linezolid, vancomycin, nitrofurantoin, trimethoprim-sulfamethoxazole, and trimethoprim. Multi-resistant *S. aureus* were isolated from samples from hypermarkets HA and HB and supermarkets SD, SE, SF, and SG.

The phenotype of antibiotic resistance of coagulase negative staphylococci isolated from turkey meat is shown in Table 10. It is worth noting that 24.56% of the coagulase-negative staphylococci isolates were multi-resistant. It should also be noted that one *S. pasteuri* strain showed resistance to 12 antibiotics: mupirocin, penicillin, lincomycine, erythromycin, tetracycline, clindamycin, streptomycin, sulfadiazine, cefoxitin, amikacin, tobramycin, and ceftaroline. All the *S. hyicus*, *S. simulans*, and *S. xylosus* isolates were susceptible to all the antimicrobials tested. Multi-resistant strains were observed in the following staphylococci species: *S. capitis* (100%), *S. cohnii* (100%), *S. epidermidis* (25%), *S. lentus* (100%), *S. pasteuri* (100%), *S. saprophyticus* (28.57%), and *S. sciuri* (50%). Resistance to mupirocin was observed in 17.54% of the coagulase-negative staphylococci. Multi-resistant coagulase-negative staphylococci were isolated from all the retailers evaluated except supermarket SF.

Neither *Campylobacter* spp. nor *Clostridium perfringens* was detected in any sample.

## 4. Discussion

We observed mesophile counts of 5.10 ± 1.36 log CFU/g in turkey meat. Jaber et al. [17] found higher counts in turkey meat from Moroccco (6.44 log CFU/g). Lower counts were reported by Augustyńska-Prejsnar et al. [34]. (4.25 ± 0.07 log CFU/g). It should be noted that poultry spoilage occurs when mesophile counts reach 8–9 log CFU/g [35], populations that were not reached in the present study. The bacterial load on poultry meat is influenced by the physiological conditions of animals at slaughter, as well as by processing, distribution, and storage circumstances [3].

*Pseudomonas* spp., lactic acid bacteria, *Brochothrix thermosphacta*, *Acinetobacter* spp., *Enterobacteriaceae*, *Staphylococcus* spp., and *Enterococcus* spp. are frequent bacteria found in poultry meat [7,36]. We observed that lactic acid bacteria were the dominant group in turkey meat (37.66%), followed by *B. thermosphacta* (22.94%) and *Pseudomonas* spp. (9.09%). These bacteria have been identified as the main spoilage microorganisms in poultry meat [8,14]. Other studies have found that the prevalent bacteria in chicken are *Pseudomonas* spp., *Staphylococcus* spp., *Carnobacterium* spp., *Aeromonas* spp., *Acinetobacter* spp., and *Weissella* spp. [16].

Among lactic acid bacteria, *Lactobacillus* spp., *Leuconostoc* spp., and *Carnobacterium* spp. are linked with the spoilage of fresh meat [37]. Other authors have also found *C. maltaromaticum* and *C. divergens* in fresh meat, with *C. divergens* being the dominant species, as in the present work [38]. *Carnobacterium* spp. have been associated with the spoilage of chicken meat [6,39]. We observed that *Carnobacterium* spp. represented 50.6% of lactic acid bacteria, followed by *Lactobacillus* spp. (34.5%) and *Leuconostoc* spp. (8.05%). In addition, Vihavainen et al. [39] reported that *C. maltaromaticum* and *C. divergens* were the dominant bacteria in chicken. It should be noted that *Lactobacillus* spp. has been isolated from broiler feathers and skin, while *Leuconostoc* spp. and *Carnobacterium* spp. have been isolated from the plant-processing environment [39].

As in the present study, *Raouterella* spp. has previously been found in raw turkey and chicken meat, although the earlier study found a different species, *Raouterella ornithinolytica*, instead of *Raoultella planticola* [30,34].

Among *Micrococcaceae*, *Kocuria* spp. and *Micrococcus* spp. Have often been isolated from fresh meat [40]. As in the present work, Höll et al. [6] isolated *Rothia nasicumurium* from chicken meat.

*Acinetobacter* spp. have also been isolated from chicken carcasses; their presence is related to cross-contamination during processing [41]. *A. johnsonii*, *A. lwoffii*, and *A. guillouiae* have been detected in chicken [42]. *Chryseobacterium* spp. has also been isolated from chicken [12,43]. *Psychrobacter* spp. was previously reported in chicken meat [16,40,43]. *Brevundimonas diminuta* was isolated from pork meat [44]. The isolation of *B. diminuta* may be of concern, as this bacterium is considered an emerging pathogen and an important multidrug-resistant microorganism [45]. In addition, *Sterophonomas* spp. and *Waurtersiella* spp. have been isolated from fresh meat [40,43].

As in the present work, Höll et al. [6] isolated *Microbacterium* spp. and *Rhodococcus* spp. from chicken meat. In addition, *Bacillus* spp. has been isolated from fresh meat [40]. In the present work, *M. maritypicum* and *R. erythropolis* were only isolated from the sample in which doxycycline was detected. These bacteria have been reported for their antimicrobial resistance [46,47]. Our results suggest that the presence of doxycycline may influence meat microbiota. It should be noted that tetracyclines are usually administered intramuscularly to food-producing animals, having an extended mean residence time in muscles, and consequently there is an extended withdrawal period for these antibiotics [48]. A study found that antimicrobial levels in muscles decreased as the withdrawal period moved forward [48]. Therefore, although the amounts of doxycycline detected in the positive meat sample were low (below the MRLs), large amounts would be present in early stages and could affect animal microbiota, which could be a source of contamination of meat.

Higher *Pseudomonas* spp. counts have been identified by Augustyńska-Prejsnar et al. [34] in turkey meat (4.29 ± 0.05 log CFU/g) compared to the counts observed in the present research (3.17 ± 0.87 log CFU/g). *Pseudomonas* spp. are important spoilage bacteria. Some species, such as *P. fluorescens*, *P. fragi*, *P. lundensis*, and *P. putid*, are often found in spoiled meat [40]. Some authors have reported that *P. putida* was the most common *Pseudomonas* spp. isolated from turkey meat, but this species was not isolated in the present work [34]. It is worth noting that *P. putida* has often been isolated from spoiled meat [40]. On the other hand, in the present work the dominant flora was *Carnobacterium* spp. rather than *Pseumdomonas* spp. Similarly, *Pseudomonas* spp. has been isolated from chicken by other authors [13,49]. Kačániová et al. [49] also isolated *P. brenneri*, *P. proteolytica*, and *P. fluorescens* from chicken meat. Oakley et al. [13] also reported the presence of the following *Pseudomonas* spp. in chicken: *P. libanensis*, *P. extremorientalis*, *P. antarctica*, *P. veronii*, *P. synxantha*, *P. marginalis*, *P. cedrina*, *P. koreensis*, *P. brenneri*, and *P. trivialis*. The presence of *P. orientalis* has also been found in meat by other authors [50]. We also isolated other *Pseudomonas* spp., including *P. rhodesiae*, *P. azotoformans*, and *P. kilorensis*. A total of 16 different species of *Pseudomonas* were identified in the present study. Kačániová et al. [49] isolated nine different *Pseudomonas* spp. from chicken meat. It is worth noting that the main contamination source of *Pseudomonas* spp. is the processing environment [11].

Enterococci counts below 1 log CFU/g were observed in 12 samples (23.53%). The other 39 samples (76.47%) showed counts between 1.30 log CFU/g and 3.28 log CFU/g. Other authors have reported that enterococci counts are usually present in raw meat at levels between 2–4 log CFU/g [51]. The dominant enterococci found in the present work was *E. faecium*. However, other authors reported that the predominant enterococci in turkey is *E faecalis* [52]. Moreover, Aslam et al. [53] did not isolate either *E. faecium* or *E. hirae* from turkey meat. Enterococci are often contaminants of poultry meat [20]. Turkey meat may become contaminated with *E. faecium* and *E. faecalis* at slaughter. As enterococci are commensals in the gut of poultry, the contamination of carcasses by fecal bacteria can occur if hygienic standards are low [20].

We isolated *S. gallolyticus*, a non-enterococcal group *D streptococci*, from 12 samples (23.53%) [54]. This bacterium has been previously reported in turkey feces [55]. As far as we know, there are no previous works on its presence in turkey meat. The isolation of *S. gallolyticus* may be of concern because this bacterium is an opportunistic pathogen in humans and can cause bacteremia, meningitis, and endocarditis [56]. In addition, the presence of this species has been linked to colon cancer in humans [52].

The presence of *Enterobacteriaceae* in fresh meat is of particular relevance, since some species are pathogens for humans [57]. In addition, these bacteria have a high deteriorating potential [57]. Higher *Enterobacteriaceae* counts in turkey meat have been reported by Augustyńska-Prejsnar et al. [34] (3.96 ± 0.03 log CFU/g, compared to 3.18 ± 1.00 log CFU/g in the present study). Augustyńska-Prejsnar et al. [34] observed that the most frequently *Enterobacteriaceae* isolated in raw turkey was *Enterobacter cloacae*, followed by *Hafnia alvei* and *Pantoea agglomerans*. In contrast, we observed that *Serratia liquefaciens* was the dominant species, followed by *Hafnia alvei* and *Escherichia coli*. Our findings that *Serratia* spp. is the dominant *Enterobacteriaceae* agrees with others studies in chicken and turkey meat [6,58]. As in the present work, Höll et al. [6] pointed out that the dominant *Enterobacteriaceae* in chicken meat were *Serratia* spp. However, they reported that the largest part of the genus *Serratia* was represented by *S. proteomaculans* rather than *S. liquefaciens*, as was observed in the present work. In addition, *S. fonticola* has been isolated from poultry [59]. Other authors have also found *Kluyvera intermedia*, *Klebsiella pneumoniae*, and *K. oxytoca* in fresh turkey meat [25,34]. *Rahnella aqualis* has also been isolated from chicken meat [40]. This species has been linked to the spoilage of pork meat [60]. *Buttiauxella warmboldiae and B gavininae* have also been isolated from chicken meat [49]. The presence of *B. agrestis* in fresh meat has also been reported by other authors [40]. *Buttiauxella* spp. has been associated with meat spoilage [57]. *Enterobacter* spp. has been found in chicken meat by other authors [49]. *Moellerella wisconsensis* may cause infections in humans [61]. This bacterium has been isolated from wild birds [62] but its presence in turkey meat has not been previously reported. We only isolated *E. coli* (40%) and *K. pneumoniae* (60%) from MacConkey agar in the meat sample in which doxycycline was detected. *K. pneumoniae* was not isolated from MacConkey agar in any other sample. Our results suggest that the presence of doxycycline could influence the *Enterobacteriaceae* species, which is dominated by *E. coli* and *K. pneumoniae*. As mentioned above, although the amounts of doxycycline detected in the positive meat sample were low (below the MRLs), large amounts would be present in early stages and could affect the animal microbiota, which could be a source of contamination of meat. Further studies are needed to confirm these findings, as there have been a limited number of samples with antibiotic residues.

As in the present work, other researchers have observed a high prevalence of *E. coli* in turkey meat [17,24]. We observed that 45.4% of the turkey samples showed positive results in chromID ESBL, a lower percentage than that reported by Díaz-Jiménez et al. [25] (84%). The use of the abovementioned medium allows detecting ESBL producers when they are present at low concentrations—particularly *E. coli*, which is one of the most common ESBL producers [23]. This fact can explain that none of the *E. coli* isolates obtained from MacConkey agar showed the ESBL phenotype, although some of the isolates were obtained from samples that were positive in chromID ESBL.

We isolated both ESBL-producing *K. pneumoniae* and ESBL-producing *E. coli* from the doxycycline positive sample. It should be noted that the *K. pneumoniae* isolates obtained from MacConkey agar in that positive sample were also the ESBL-producing phenotype. These findings suggest that doxycycline may promote the presence of ESBL-producing *K. pneumoniae*. In fact, some studies indicate that the use of tetracyclines requires attention, due to the development of the antimicrobial resistance of *K. pneumoniae* and *E. coli* [63]. Like Díaz-Jiménez et al. [25], we did not isolate any carbapenemase-producing *Enterobacteriaceae* from chromID CARBA SMART.

We observed that *E. coli* isolates from turkey meat showed higher resistance rates than those reported by Díaz-Jiménez et al. [25] for ampicillin (100% vs. 90.2%), while lower rates were found for trimethoprim- sulfamethoxzoale (17.39% vs. 53.7%) and ciprofloxacin (52.17% vs. 53.7%). Higher rates of resistance than those found in the present work have been reported for *E. coli* isolates from poultry meat for nalidixic acid (60.7% vs. 43.48% in the present work) and gentamicin (19% vs. 0% in the present work), while lower rates of resistance were found for doxycycline (29.8% vs. 65.22% in the present work) [25].

We found high resistance rates to aztreonam (91.3%) in *E. coli* isolates recovered from chromID ESBL. This finding is relevant, as aztreonam is categorized as “Category A: antimicrobial to avoid” in animals [64]. In addition, we observed high resistance rates to fluoroquinolones and cephalosporins of the third or fourth generation. Further, resistance to colistin was observed (8.69%). It should be pointed out that fluoroquinolones, cephalosporins of third or fourth generation, and colistin have been categorized as “Category B: antimicrobials to restrict” in animals [64].

Díaz-Jiménez et al. [25] also observed that all the ESBL-producing *K. pneumoniae* recovered from chromID ESBL were multi-resistant with high resistance rates to ampicillin, cefotaxime, ciprofloxacin, trimethoprim-sulfamethoxazole, and doxycycline (above 60%). Higher resistance rates were reported by Díaz-Jiménez et al. [25] for tigecycline (62.3% vs. 25% in the present work) and lower rates were reported for aztreonam (35.7% vs. 87.5) and ceftazidime (28.6% vs. 75%).

We observed high resistance rates to aztreonam (87.3%) in *K. pneumoniae* isolates. In addition, resistance to tigecycline was observed (25%). Both aztreonam and tigecycline are categorized as “Category A: antimicrobial to avoid” in animals [64]. Moreover, we found high resistance rates to fluoroquinolones and cephalosporins of the third or fourth generation. In addition, resistance to colistin was observed (50%). As mentioned above, fluoroquinolones, cephalosporins of the third or fourth generation and colistin have been categorized as “Category B: antimicrobials to restrict” in animals [64]. *K. pneumoniae* is an opportunistic pathogen that is capable of persisting in various reservoirs, including hospitals, livestock, wastewater, and meat [65,66]. Therefore, the high resistance rates to critical antimicrobials are of special concern.

Staphylococci are frequent inhabitants of the mucous membranes and skin [67] and of the chicken intestinal tract [65]. Among the species of the genera *Staphylococcus*, there are both commensals and pathogens. *S. aureus* is a recognized foodborne pathogen [9]. Other *Staphylococcus* spp., such as *S. epidermidis*, *S. intermedius*, *S. saprophyticus*, *S. hyicus*, *S. pasteuri*, *S. cohnii*, *S. warneri*, *S. lugdunensis*, *S. sciuri*, and *S. simulans*, can cause infections in humans [21,68,69,70,71,72,73]. Some of these staphylococci were isolated in the present work (*S. aureus*, *S. epidermidis*, *S. saprophyticus*, *S. hyicus*, *S. pasteuri*, *S. cohnii*, *S. warneri*, *S. sciuri*, and *S. simulans*). Other authors have also isolated *S. aureus*, *S. epidermidis*, *S. pasteuri*, *S. warneri*, and *S. capitis* from chicken meat and chicken carcasses [12,20,40]. *S. saprophyticus*, *S. cohnii*, *S. warneri*, *S. lentus*, *S. simulans*, *S. sciuri*, and *S. xylosus* have been isolated from chickens at farm level [67,74]. We also detected other species in turkey meat: *S. intermedius*, *S. hyicus*, *S. equorum*, *S. vitulinus*, *S. fleurettii*, and *S. sciuri*. We isolated 16 different species of *Staphylococcus*, as well as *Macrococcus caseolyticus*. The genera *Macrococcus* belongs to the family *Staphylococcaceae* and is closely linked to the genera *Staphylococcus* [75]. Currently, there is special interest in *M. caseolyticus* because of its potential for disseminating antimicrobial resistance [76]. In addition, this bacterium has been isolated from pork and beef meat [75]. We found a *M. caseolyticus* isolate resistant to 11 antibiotics, including antimicrobials of “Category A: antimicrobial to avoid” (mupirocin, linezolid. rifampicin, and vancomycin), and “Category B: antimicrobials to restrict” (cephalosporins of third or fourth generation) [64]. This finding is of special concern because of the isolate’s potential to disseminate antimicrobial resistance [76].

Certain strains of *S. aureus* can produce enterotoxins, and the consumption of foods containing the preformed toxins can cause a foodborne illness. In addition, there is a serious concern about the occurrence of MRSA in meat and poultry [36]. A similar prevalence of *S. aureus* has been reported in turkey meat by Hanson et al. [77]. (19.4%, 16.7% in the present work), while other authors have reported a higher prevalence 35.3–77% [27,78]. Some authors have not detected any MRSA in turkey meat, while others reported a prevalence between 3.85% and 35.3% [27,78]. We detected MRSA in 7.84% of the turkey meat samples. The contamination of poultry meat with *S. aureus* can be of animal or human origin, as contamination by handlers can occur [20].

As in the present work, other researchers have reported that *S. aureus* isolated from turkey meat showed high resistance rates against erythromycin, cefoxitin, tetracycline, clindamycin, and ciprofloxacin, while lower resistance rates were observed for chloramphenicol and gentamicin [79,80]. In addition, Kraushaar et al. [79] observed that all *S. aureus* isolated from turkey were susceptible to linezolid and vancomycin.

We observed that 11.11% of the *S. aureus* isolates showed resistance to rifampicin and mupirocin antimicrobials included in “Category A: antimicrobial to avoid” [64]. Other authors have also found resistance to mupirocin and rifampicin in *S. aureus* isolated from turkey, but at lower levels than those found in the present study (3.4%) [77]. We also observed resistance to antimicrobials included in Category B (66.67% showed resistance to fluoroquinolones).

We observed that only 14.04% of the coagulase-negative staphylococci (CNS) presented susceptibility to all the antimicrobials tested. Pyzik et al. [81] also observed that a relatively high percentage of CSN strains isolated from poultry showed multi-resistance (30.71% vs. 24.56% in the present work), with resistance above 30% for penicillin and tetracycline [81]. We isolated a *S. pasteuri* strain that was resistant to 12 antibiotics: mupirocin, penicillin, lincomycine, erythromycin, tetracycline, clindamycin, streptomycin, sulfadiazine, cefoxitin, amikacin, tobramycin, and ceftaroline. Other authors have also isolated multi-resistant *S. pasteri* from pheasant meat, which showed resistance to penicillin, oxacillin, gentamicin, tetracycline, and erythromycin [82]. Moreover, in the current study, resistance to mupirocin was observed in 17.54% of the CNS isolates, an antimicrobial included in Category A [64]. These findings are of concern, as coagulase-negative staphylococci could be a reservoir of clinically relevant resistant genes that could be transferred to *S. aureus* isolates [83].

Like Mezher et al. [84], we did not isolate any *Campylobacter* spp. in turkey meat. However, other studies have shown a high prevalence of *Campylobacter* spp. in turkey meat [85]. Narvaez et al. [86] found *Campylobacter* spp. in 14.2% of the turkey samples.

*Clostridium perfringens* was not detected in the present work; few works deal with the detection of this pathogen in poultry meat, indicating populations of 1.0–1.2 log CFU/g [87].

In total, 35 different genera were identified in the present work, a higher number than that identified by Kačániová et al. [40] in chicken meat (15 genera). In addition, we detected some species that are considered as opportunistic pathogens and others that are recognized foodborne pathogens.

## 5. Conclusions

This study emphasized that turkey meat microbiota can be a source of both recognized foodborne pathogens and opportunistic or emerging pathogens. Moreover, turkey meat can be a source of *K. pneumoniae*, *E. coli*, *S. aureus*, coagulase negative staphylococci, and *M. caseolyticus* resistance to critical antibiotics, according to European Medicine Agency (EMA) criteria.

The presence of multi-resistant bacteria in turkey meat is of particular concern, and special measures should be taken within the framework of the One Health approach.

## Figures and Tables

**Table 1:** Percentage and number of isolates identified in turkey samples from Plate Count agar.

Microbial Group and Species	Number of Isolates	Percentage (%)
**Lactic acid bacteria** *Lactobacillus* spp. (30) * Carnobacterium divergens (31) *Carnobacterium maltaromaticum* (13) *Lactococcus lactis* (5) *Lactococcus raffinolactis* (1) *Leuconostoc mesenteroides* (4) *Leuconostoc carnosum* (2) *Leuconostoc citreum* (1)	87	37.66%
***Brocchotrix thermosphacta***	53	22.94%
***Pseudomonas* spp.** *P. fragi* (10) *P. lundensis* (4) *P. brenneri* (2) *P. libanensis* (2) *P. fluorescens* (1) *P. extremorientalis* (1) *P. taetrolens* (1)	21	9.09%
***Enterobacteriaceae*** *Serratia liquefaciens* (5) *Serratia proteamaculnas* (5) *Serratia marcescens* (1) *Escherichia coli* (2) *Hafnia alvei* (2) *Ewingella americana* (1) *Raoultella planticola* (1 *Rahnella inusitata* (1) *Erwinia rhapontici* (1))	19	8.23%
***Microccaceae*** *Kocuria varians* (5) *Kocuria salsicia* (4) *Kocuria rizhophila* (1) *Staphylococcus saprophyticus* (3) *Staphylococcus warneri* (1) *Macrococcus caseolyticus* (2) *Micrococcus luteus* (1) *Rothia nasimurium* (1)	18	7.79%
**Enterococci** *Enterococcus faecalis* (3)	3	1.30%
**Other Gram-negative bacteria** *Chryseobacterium scophtalnum* (4) *Chryseobacterium aquaticum* (1) *Chryseobacterium rhizosphaerae* (1) *Chryseobacterium piscium* (1) *Chryseobacterium sigense* (1) *Acinetobacter gulliouiae* (2) *Acinetobacter lwoffii* (2) *Acinetobacter johnsonii* (1) *Acinetobacter harbonensis* (1) *Brevundimonas diminuta* (1) *Stenotrophomonas rhizophila* (5) *Wautersiella falsenii* (2) *Psychrobacter pulmonis* (1)	23	9.96%
**Other Gram-positive bacteria** *Microbacterium liquefaciens* (3) *Microbacterium maritypicum* (2) *Rhodococcus erythropolis* (1) *Bacillus endophyticus* (1)	7	3.03%
Total	231	100

* Number of isolates.

**Table 2:** Percentage and number of *Pseudomonas* spp. isolated from chromogenic agar for *Pseudomonas* in turkey samples.

Species	Number of Isolates	Percentage (%)
*Pseudomonas libanensis*	31	31
*Pseudomonas extremorientalis*	14	14
*Pseudomonas fluorescens*	12	12
*Pseudomonas antarctica*	7	7
*Pseudomonas rhodesiae*	6	6
*Pseudomonas azotoformans*	5	5
*Pseudomonas veronii*	5	5
*Pseudomonas brenneri*	5	5
*Pseudomonas orientalis*	3	3
*Pseudomonas synxantha*	3	3
*Pseudomonas marginalis*	2	2
*Pseudomonas cedrina*	2	2
*Pseudomonas trialis*	2	2
*Pseudomonas kilorensis*	1	1
*Pseudomonas proteolítica*	1	1
*Pseudomonas koreensis*	1	1
Total *Pseudomonas* spp.	100	100

**Table 3:** Number and percentage of enterococci isolated from Kanamycin Esculin Azide agar in fresh turkey meat.

Specie	Number of Isolates	Percentage (%)
*E faecium*	16	38.10
*E. faecalis*	10	23.81
*E. gallinarum*	7	16.67
*E gilvus*	5	11.90
*E. cassiliflavus*	3	7.14
*E. hirae*	1	2.38
Total *enterococci*	42	100

**Table 4:** Percentage and number of *Enterobacteriacceae* isolates identified in turkey samples from MacConkey agar.

Specie	Number of Isolates	Percentage (%)
*Serratia liquefaciens*	22	16.42
*Hafnia alvei*	19	14.18
*Escherichia coli*	19	14.18
*Ewingella americana*	18	13.43
*Buttiauxella gaviniae*	16	11.94
*Rahnella aquatilis*	8	5.97
*Serratia proteamaculans*	7	5.22
*Buttiauxella warmboldiae*	5	3.73
*Buttiauxella agrestis*	4	2.99
*Serratia fonticola*	4	2.99
*Klebsiella pneumoniae*	3	2.24
*Klebsiella oxytoca*	2	1.49
*Moellerella wisconsensis*	2	1.49
*Kluywera intermedia*	2	1.49
*Enterobacter cloacae*	1	0.75
*Pantoea aglomerans*	1	0.75
*Yersinia enterocolitica*	1	0.75
Total *Enterobacteriacceae*	134	100

**Table 5:** Antimicrobial resistance phenotype of multi-resistant *E. coli* isolated from turkey meat.

Medium of Isolation (Number of Isolates)	Antibiotic Resistance Phenotype ^1^ (Number of Isolates)	Retailer ^3^
ChromID ESBL (23)	TE-S-ENR-CIP-NA-PRL-AMP (1)	SA
	TE-S-PRL-CT-K-SUZ-SXT-W (1)	SC
	ENR-PRL-AMP-ATM-CAZ-CPD-CTX-CRO (1)	SD
	TE-PRL-AMP-ATM-CPD-CTX-CRO-DO (1)	HB
	AMP-ATM-CAZ-CPD-CTX-CRO-DO S (1)	SA
	TE-S-SUZ-PRL-AMP-ATM-CTX- CRO-C (1)	HA
	TE-S-PRL-AMP-ATM-CAZ-CPD-CTX-CAZ-CRO-DO K (1)	SE
	TE-S-PRL-AMP-ATM-CAZ-CPD-CTX-CRO-DO (1)	SE
	TE-S-PRL-AMP-ATM-CAZ-CPD-CTX-CRO-DO-SUZ (1)	HA
	TE-S-ENR-AMP-SUZ-ATM-CAZ-CPD-CTX-CRO-SXT-W (1)	SG
	TE-S-ENR-AMP- PRL-ATM-CAZ-CPD-CTX-CRO-DO (1) ^2^-	SF
	TE-S-PRL-ENR-CIP-AMP-ATM-CAZ-CPD-CTX-CRO-DO (1)	SE
	ENR-CIP-NA-PRL-AMP-ATM-CTX-CPD-CAZ-CRO-GAT-LEV-NOR (1)	SF
	AMP-ATM-CTX-CAZ-CPD-CTX-CRO-C-CIP-ENRO-NA-PRL-S-SUZ (1)	SG
	TE-S-ENR-CIP-PRL-AMP-SUZ-ATM-CAZ-CPD-CTX-CRO-DO-C (1)	HB
	TE-S-ENR-CIP-NA-PRL-AMP-SUZ-ATM-CAZ-CPD-CTX-CRO-DO-C-LEV (1)	HB
	TE-S-ENR-CIP-NA-PRL-AMP-SUZ-ATM-CAZ-CPD-CTX-CRO-DO-LEV-NOR (1)	HA
	TE-S-ENR-CIP-NA-PRL-AMP-ATM-CAZ-CPD-CTX-CRO-DO-GAT-LEV-NOR-(1)	SA
	TE-S-ENR-CIP-NA-PRL-K-SUZ-AMP-ATM-CAZ-CPD-CTX-CRO-DO-MH (1)	SB
	TE-S-ENR-CIP-NA-PRL-AMP-CT-SUZ-SXT-W-ATM-CTX-CRO-DO-LEV-NOR (1)	SC
	TE-S-ENR-CIP-NA-PRL-AMP--SUZ-SXT-W-ATM-CPD-CTX-CRO-LEV-NOR-FEP (1)	HB
	TE-S-ENR-CIP-NA-PRL-AMP-SUZ-ATM-XAZ-CPD-CTX-CRO-DO-LEV-NOR-GAT (1)	HB
	TE-S-ENR-CIP-NA-PRL-AMP-SUZ-CAZ-CPD-CRO-DO-C-LEV-NOR-FEP-GAT (1)	SA
MacConkey agar (10)	S-ENR-PRL-DO (1)	SG
	S-ENR-NA-PRL-AMP (1)	SB
	TE-AMP-CT-DO-F-AUG (1)	HA
	TE-S-PRL-AMP-SUZ-SXT-DO (1) ^2^	SF
	TE-S-PRL-AMP- SUZ-SXT-W-DO (1)	SA
	TE-S-PRL-AMP-SUZ-SXT-W-DO (1)	SE
	TE-S-AMP-K-SUZ-SXT-W-DO-C-MH-CN (1)	SC
	TE-S-ENR-CIP-NA-CT-K-SUZ-DO-C-LEV-NOR (1)	HB
	TE-ENR-CIP-NA-PRL-AMP-SUZ-DO-C-LEV-GAT (1)	SG
	TE-S-ENR-CIP-NA-PRL-AMP-SUZ-SXT-W-DO-MH-(1)	SE

^1^ TE: tetracycline; S: streptomycin; ENR: enrofloxacin; CIP: ciprofloxacin; NA: nalidixic acid; PRL: piperacillin; PRL: piperacillin; AMP: ampicillin, CT: colistine, K: kanamycin, SUZ: sulfadiazine, SXT: trimethoprim-sulfamethoxazole, W: trimethoprim; ATM: aztreonam; CAZ: ceftazidime, CPD: cefpodoxime, CTX: cefotaxime, CRO: ceftriaxone; DO: doxycycline; C, chloramphenicol; GAT: gatifloxacin; LEV: levofloxacin; NOR:, levofloxacin; MH: minocycline; FEP: cefepime; F: nitrofurantoin; AUG: amoxicillin-clavulanate; CN: gentamicin; ^2^ strain isolated from the sample meat containing antibiotic residues; ^3^ hypermarket (HA, HB), supermarket (SA, SB, SC, SD, SE, SF, SG), traditional shop (TA).

**Table 6:** Antimicrobial resistance phenotype of *Klebsiella* spp. isolated from turkey samples.

Species (Number of Isolates)	Antibiotic Resistance Phenotype ^1^ (Number of Isolates)	Retailer ^6^
*Klebsiella oxytoca* (2)	CT-AMP ^2^	HA
	AMP ^2^	TA
*Klebsiella pneumoniae* (8)	CT-AMP-PRL-FEP-CAZ-CPD-CTX-CRO-ATM-ENRO-CIP-NA-SUZ-SXT-W-S (1) ^2,3,4^	SF
	CT-AMP-PRL-FEP-CAZ-CPD-CTX-CRO-ATM-ENRO-CIP-SUZ-SXT-W-S-K-TOB-TE-DO (1) ^2,3,4^	SF
	CT-AMP-PRL-FEP-CAZ-CPD-CTX-CRO-ATM-ENRO-CIP-NA-SUZ-SXT-W-S- K-TOB-TE-DO-F (1) ^2,3,4^	SF
	AMP-PRL-FEP-CPD-CTX-CRO-ATM-ENRO-CIP-SUZ-SXT-W-S-K-TOB-TE (2) ^3,4,5^	SF
	AMP-PRL-FEP-CAZ-CPD-CTX-CRO-ATM-ENRO-CIP-SUZ-SXT-W-S- K-TOB-TE-DO-F-TGC-LEV-NOR (1) ^3,4,5^	SF
	CT-AMP-PRL-CAZ-CPD-CRO-ENRO-CIP-SUZ-S-TE-DO-F-TGC-SAM-FOX-ETP-AK-MH (1) ^4,5^	SA
	AMP-PRL-FEP-CAZ-CPD-CTX-CRO-ATM-ENRO-CIP-SUZ-SXT-W-S- K-TOB-TE-DO (1) ^4,5^	HA

^1^ CT: colistine, AMP: ampicillin, PRL: piperacillin, FEP: cefepime, CAZ: ceftazidime, CPD: cefpodoxime, CTX: cefotaxime, CRO: ceftriaxone, ATM: aztreonam, ENR: enrofloxacin, CIP: ciprofloxacin, NA: nalidixic acid, SUZ: sulfadiazine, SXT: trimethoprim-sulfamethoxazole, W: trimethoprim, S: streptomycin, K: kanamycin, TOB: tobramycin, TE: tetracycline, DO: doxycycline, F: nitrofurantoin, SAM: ampicillin + surfabactam, FOX: cefoxitin, TGC: tigecycline; ETP: ertapenem, AK; amikacin, MH: minocycline.); ^2^ strain isolated from MacConkey agar; ^3^ strain isolated from the sample meat containing antibiotic residues; ^4^ strain showing ESBL phenotype; ^5^ strain isolated from ESBL chromogenic agar; ^6^ hypermarket (HA, HB), supermarket (SA, SB, SC, SD, SE, SF, SG), traditional shop (TA).

**Table 7:** Number and percentage of *Staphylococcus* spp. *and Macrococcus* spp. isolates identified in turkey samples recovered from mannitol salt agar.

Species	Number of Isolates	Percentage (%)
*Staphylococcus saprophyticus*	39	31.45
*Staphylococcus equorum*	17	13.7
*Macrococcus caseolyticus*	15	12.1
*Staphylococcus aureus*	10	8.1
*Staphylococcus epidermidis*	8	6.45
*Staphylococcus vitulinus*	8	6.45
*Staphylococcus lentus*	5	4.03
*Staphylococcus cohnii*	4	3.23
*Staphylococcus warneri*	4	3.23
*Staphylococcus xylosus*	4	3.23
*Staphylococcus fleurettii*	3	2.41
*Staphylococcus pasteuri*	2	1.61
*Staphylococcus sciuri*	2	1.61
*Staphylococcus capitis*	1	0.8
*Staphylococcus hyicus*	1	0.8
*Staphylococcus simlulans*	1	0.8
Total	124	100

**Table 8:** Antimicrobial resistance phenotype of *Macrococcus caseolyticus* from turkey samples.

Species (Number of Isolates)	Antibiotic Resistance Phenotype ^1^ (Number of Isolates)	Retailer ^3^ (Number of Isolates)
*Macrococcus caseolyticus* (8)	susceptible to all antibiotics tested (4) ^2^	SB (3) SC (1)
	FOX (1) ^2^	SC (1)
	MY (1) ^2^	SA (1)
	PUM (1) ^2^	SB (1)
	MY-PUM-FAD-LZD-P-RD-TZD-TY-VA-ERY-CMN (1) ^2^	SE (1)

^1^ FOX: cefoxitin. MY: lincomycine, PUM: mupirocin, FAD: fusidic acid. LZD: linezolid. P: penicillin, RD: rifampicin, TZD: tedizolid, TY: ttylosin, VA: vancomycin, ERY: erythromycin. CMN: clindamycin; ^2^ strain isolated from mannitol salt agar; ^3^ hypermarket (HA, HB), supermarket (SA, SB, SC, SD, SE, SF, SG), traditional shop (TA).

**Table 9:** Antimicrobial resistance phenotype of *S. aureus* from turkey samples.

Methicillin-Resistant Isolates (Number of Isolates)	Antibiotic Resistance Phenotype ^1^ (Number of Isolates)	Retailer ^4^
No (5)	P-PNG-TE-DO (1) ^2^	SF
	P-PNG-TE-MH-ENR-NOR (1) ^2^	SG
	P-PNG-TE-ENRO-CIP-GAT-NOR-LEV (1) ^2^	SG
	P-PNG-TE-DO-ENRO-CIP-GAT-NOR-LEV- MY- TZD-TY-ERY-CMN-S (1) ^2^	HA
	P-PNG-TE-DO-ENRO-CIP-NOR-SUZ (1) ^3^	HB
Yes (4)	P-PNG-TE-DO-FAD-FOX (1) ^2^	SE
	P-PNG- TE- ENRO-CIP-GAT-NOR-LEV-MY-TY-ERY-CMN-FOX (1) ^3^	SD
	P-PNG-TE-ENRO-CIP-GAT-NOR-LEV-MY-TY-ERY-CMN-FOX-S-SUZ-AK-C-K (1) ^3^	HB
	P-PNG-TE-MH-MY-TY-ERY-CMN-FOX-S-SUZ-AK-K-PUM-TOB-CPT-CN-QD-RD-FAD (1) ^3^	HA

^1^ P: penicillin, PNG: benzilpenicillin, TE: tetracycline, DO: doxycycline, MH: minocycline, ENR: enrofloxacin, NOR: norfloxacin, CIP: ciprofloxacin, GAT: gatifloxacin, LEV: levofloxacin, MY: lincomycine. TZD: tedizolid, TY: tylosin, ERY: erythromycin. CMN: clindamycin, S: streptomycin, SUZ: sulfadiazine, FAD: fusidic acid, FOX: cefoxitin, AK: amikacin, C: chloramphfnicol, K: kanamycin, PUM: mupirocin, TOB: tobramycin, CPT: ceftaroline, CN: gentamycin, QD: quinupristin-dalfopristin, RD: rifampicin; ^2^ strain isolated from MSA; ^3^ strain isolated from MRSA; ^4^ Hypermarket (HA, HB), supermarket (SA, SB, SC, SD, SE, SF, SG), traditional shop (TA).

**Table 10:** Antimicrobial resistance phenotype of coagulase-negative staphylococci isolated from turkey samples.

Specie (Number of Isolates)	Antibiotic Resistance Phenotype ^1^ (Number of Isolates)	Retailer ^4^ (Number of Isolates)
*Staphylococcus capitis* (1)	PUM-P-ENR (1) ^2^	SD (1)
*Staphylococcus cohnii* (3)	MY-ERY-TE (1) ^2^	SF (1)
	P-MY-TE-DO-FAD (1) ^2^	HB (1)
	MY-ERY-TE-TY-CMN-C-W (1) ^2^	HB (1)
*Staphylococcus epidermidis* (4)	PUM-P (1) ^2^	HA (1)
	PUM-ERY (1) ^2^	SB (1)
	P-ERY (1) ^2^	SA (1)
	PUM-P-ERY (1) ^2^	SG (1)
*Staphylococcus equorum* (7)	susceptible to all antibiotics tested (1) ^2^	SB (1)
	ERY (4) ^2^	SC (4)
	S (1) ^2^	TA (1)
	MY-TE (1) ^2^	SC (1)
*Staphylococcus fleurettii* (1)	P-MY (1) ^2^	HB (1)
*Staphylococcus hyicus* (3)	susceptible to all antibiotics tested (1) ^2^	SG (1)
*Staphylococcus lentus* (3)	MY-TE- DO-CMN-ENRO (1) ^2^	SA (1)
	MY-TE-DO-S-CMN (1) ^2^	SC (1)
	MY-TE-DO-CMN-W-ENRO-SUZ (1) ^2^	SC (1)
*Staphylococcus pasteuri* (2)	PUM-P-MY-ERY-TE-CMN-S-SUZ FOX-AK-TOB-CPT (1) ^2^	SA (1)
*Staphylococcus saprophyticus* (21)	susceptible to all antibiotics tested (2) ^2^	HB (1) TA (1)
	ERY (1) ^2^	SF (M1)
	P (2) ^2^	SA (1) TA (1)
	FAD (1) ^2^	HA (1)
	FAD-P (2) ^2^	SC (1) TA (1)
	TE (1) ^2^	SA (1)
	TE-DO (4) ^2^	SA (2) SC (2)
	MY-TE (1) ^2^	SC (1)
	MY-TE-DO (1) ^2^	SA (1)
	P-TE-DO-FAD (1) ^2^	SA (1)
	MY-PUM-FAD-P (1) ^2^	HA (1)
	P-ERY-CMN (1) ^2^	TA (1)
	MY-PUM-TE-AK-CPT (1) ^2^	SA (1)
	MY-PUM-FAD-P-S-SUZ-AK-K-CPT (1) ^2^	SA (1)
	MY-PUM-P-TE-FOX-AK-K-CPT-CN (1) ^2^	SC (1)
*Staphylococcus sciuri* (2)	FAD (1) ^2^	SB (1)
	MY-TE-FAD (1) ^2^	SB (1)
*Staphylococcus simulans* (1)	susceptible to all antibiotics tested (1) ^2^	SG (1)
*Staphylococcus vitulinus* (4)	susceptible to all antibiotics tested (3) ^2^	HB (1)
	TE (1) ^2^	HB (1)
*Staphylococcus warneri* (4)	P-ERY (1) ^2^	SB (1)
	PUM-P (1) ^2^	TA (1)
	TOB-CN-K (2) ^2,3^	SA (1)
*Staphylococcus xylosus* (1)	susceptible to all antibiotics tested (1) ^2^	SA (1)

^1^ PUM: mupirocin, P: penicillin, ENR: enrofloxacin, MY: lincomycine, ERY: rrythromycin. TE: tetracycline, DO: doxycycline. FAD: fusidic acid, TY: tylosin, CMN: clindamycin, C: chloramphenicol, W: trimethoprim. S: streptomycin., SUZ: sulfadiazine, FOX: cefoxitin. AK: amikacin, TOB: tobramycin, CPT: ceftaroline, CN: gentamycin, K: kanamycin; ^2^ strain isolated from Mannitol Salt agar; ^3^ strain isolated from meat with presence of residues; ^4^ hypermarket (HA, HB), supermarket (SA, SB, SC, SD, SE, SF, SG), traditional shop (TA).

## Data Availability

Not applicable.
